# Supplementary material for: More frequent intense and long-lived storms dominate the springtime trend in central US rainfall
Source: Nat Commun. 2016 Nov 11;7:13429. doi: 10.1038/ncomms13429 (PMC5114602; doi:10.1038/ncomms13429)
Supplement: Supplementary Information — Supplementary Figures 1-11 [file ncomms13429-s1.pdf]

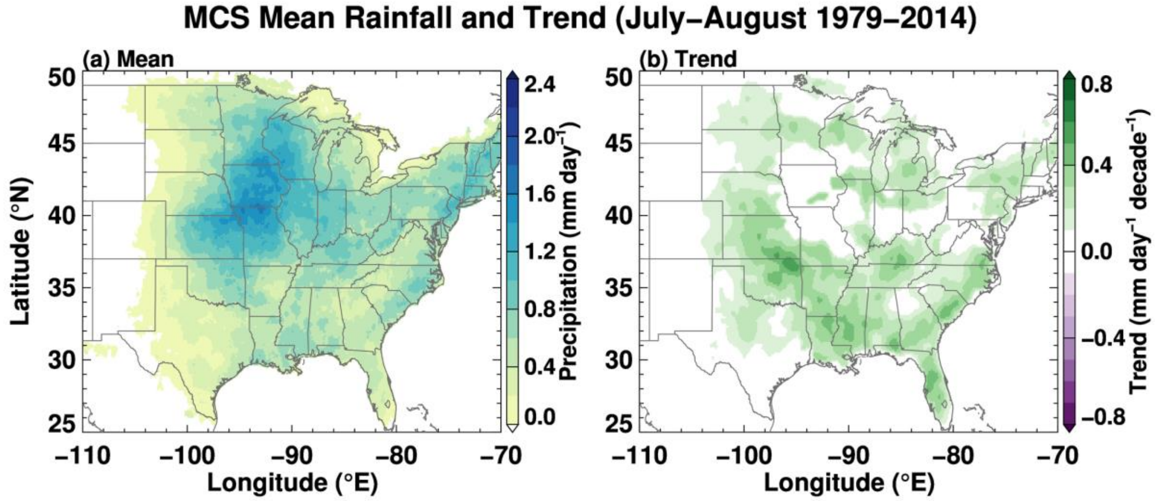

Supplementary Figure 1. Summer mesoscale convective systems rainfall climatology and trends. Mesoscale convective system (MCS) (a) mean total rainfall and (b) total rainfall trend from 1979-2014. Total rainfall shown is the accumulated MCS rainfall during July-August divided by the total number of days (62). Only trends with statistical significance above 95% using a two-tailed Student-t test are shown.

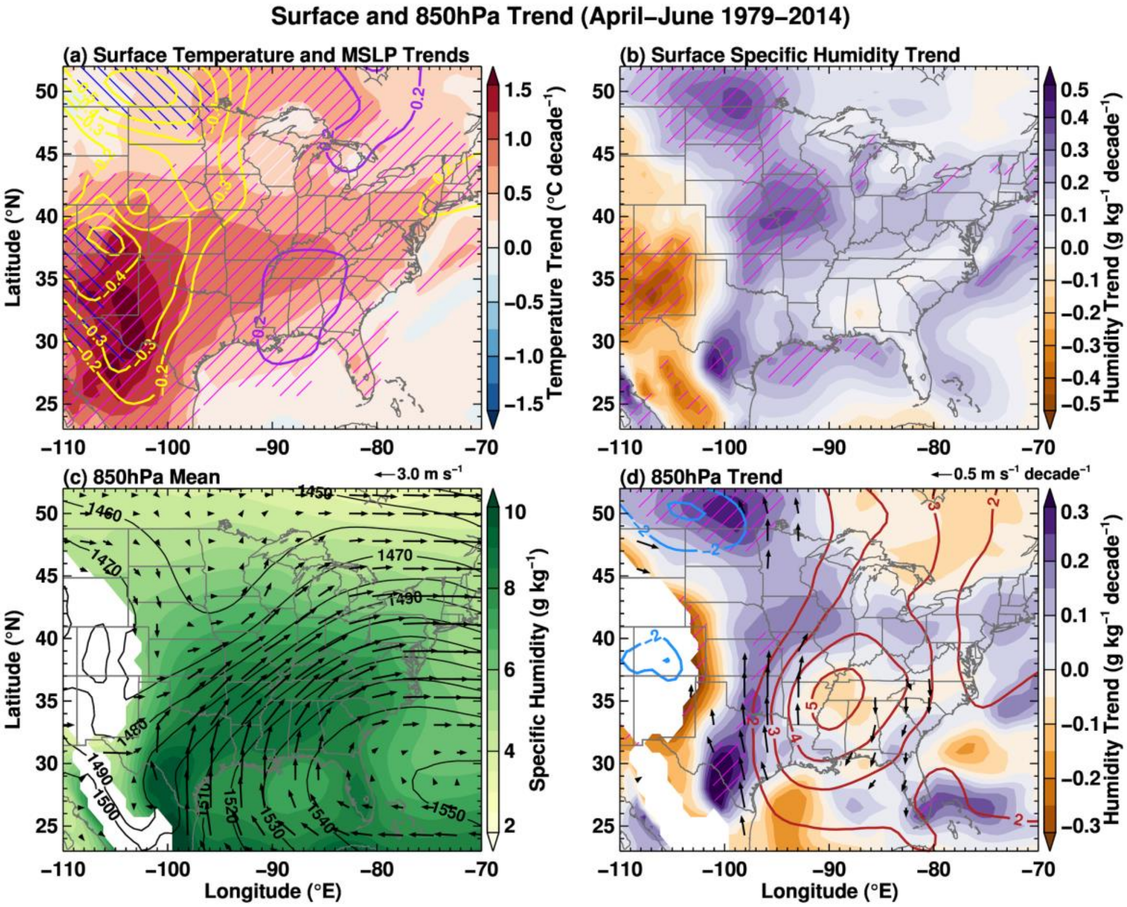

Supplementary Figure 2. Large-scale environment climatology and trends from ERA-Interim Reanalysis during occurrence of High-Precipitation mesoscale convective systems. (a) Surface temperature (shaded) and mean sea level pressure (MSLP) trends (MSLP contours in 0.1 hPa decade^{-1} intervals, purple/yellow contours denote positive/negative MSLP trends), (b) surface specific humidity trends (shaded), (c) 850 hPa mean specific humidity (shaded), geopotential height (contours, in 10 m intervals), and wind (arrows), and (d) 850 hPa trends in specific humidity (shaded), geopotential height (contours, in 1 m decade^{-1} intervals, red/blue contours denote positive/negative geopotential height trends), and wind (arrows, statistically significant at 95%). Grid points with a statistical significance exceeding the 95% confidence interval are marked by (a) pink hashes for temperature and blue hashes for MSLP, (b,d) purple hashes for specific humidity. Areas with mean surface pressure below 850 hPa are masked out in (c,d).

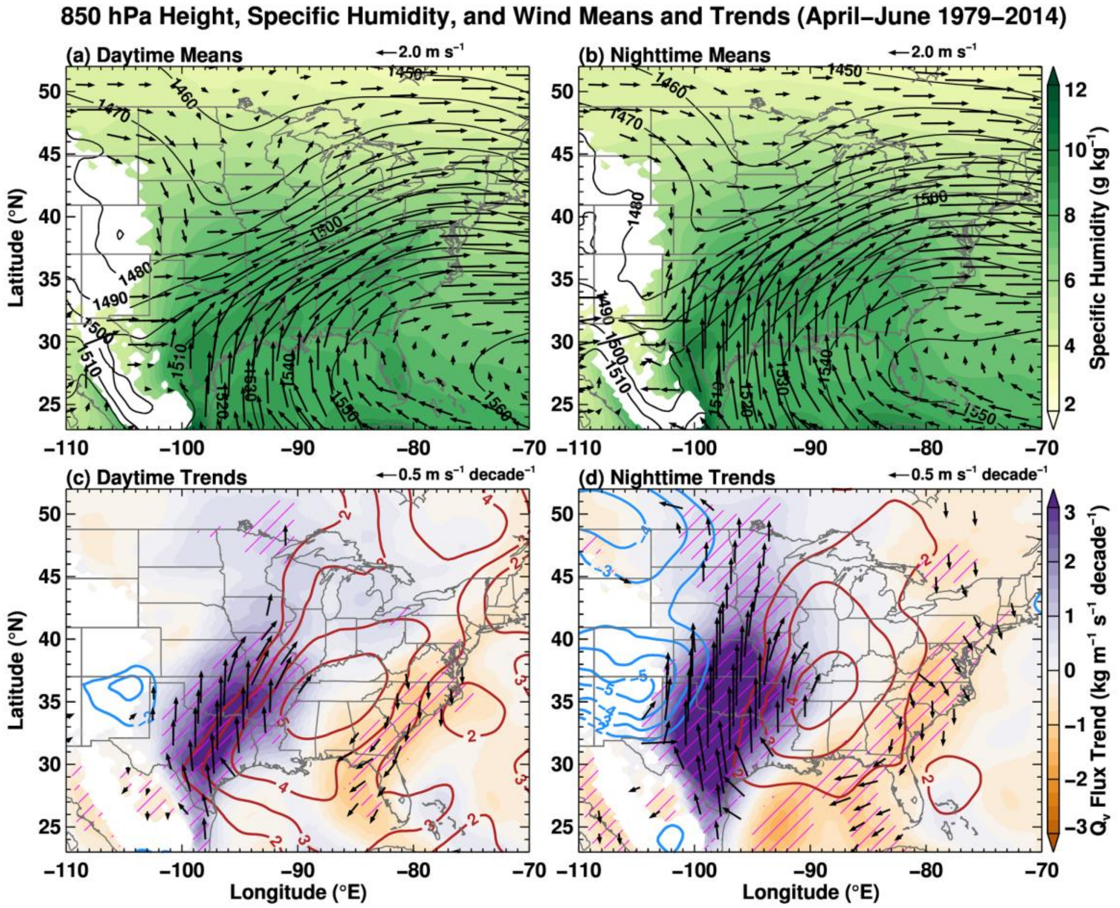

Supplementary Figure 3. 850 hPa large-scale environment climatology and trends during occurrence of High-Precipitation mesoscale convective systems. 850 hPa mean (top panels) and trends (bottom panels) composited for (a,c) daytime hours (15-24 UTC, ~09-18 LT) and (b,d) nighttime hours (03-12 UTC, ~21-06 LT). (a, b) 850 hPa mean specific humidity (shaded), geopotential height (contours, in 10 m intervals), and wind (arrows), (c, d) 850 hPa trends in meridional moisture flux (shaded), geopotential height (contours, in 1 m decade^{-1} intervals), and wind (arrows, statistically significant at 95%). Purple hashes in (c,d) denote meridional water vapor flux trends that are statistically significant at 95%.

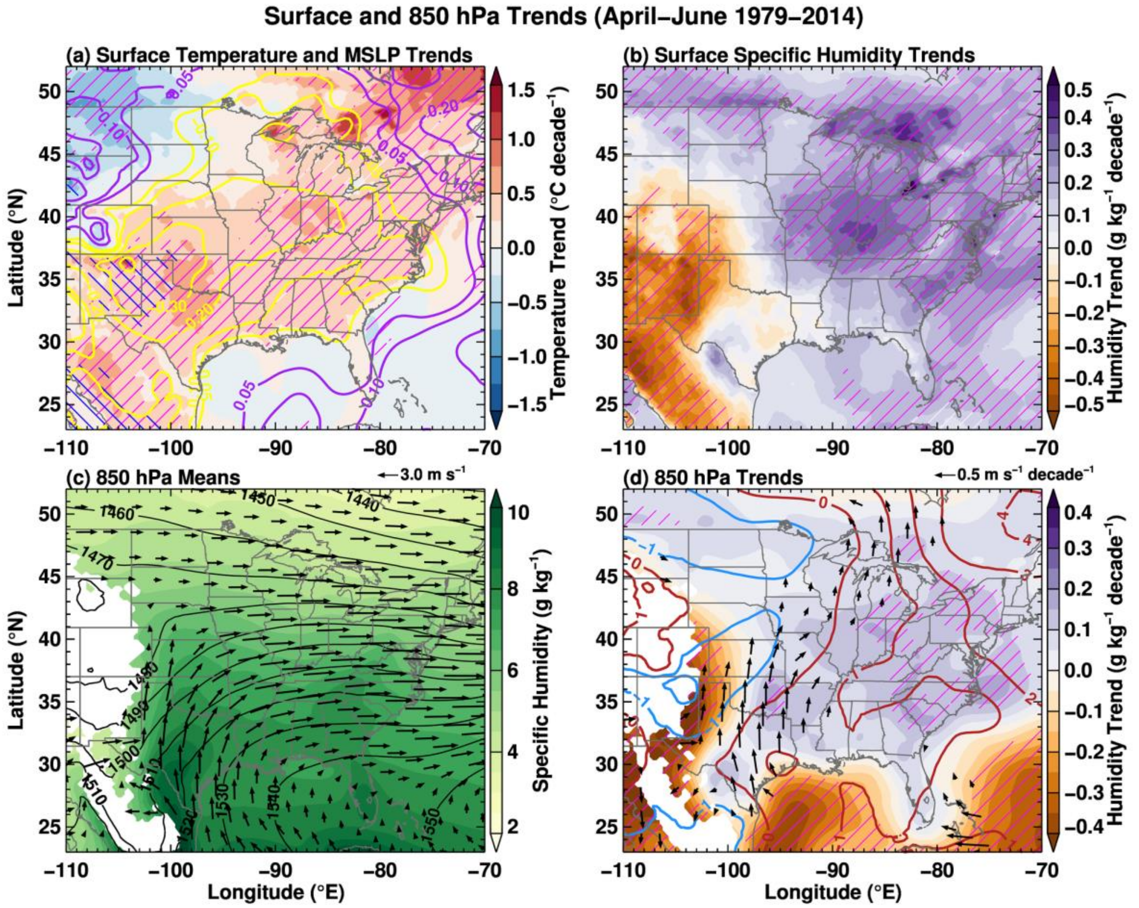

Supplementary Figure 4. Large-scale environment climatology and trends during all days in April-June. (a) Surface temperature (shaded) and mean sea level pressure (MSLP) trends (MSLP contours in 0.1 hPa decade^{-1} intervals, purple/yellow contours denote positive/negative MSLP trends), (b) surface specific humidity trends (shaded), (c) 850 hPa mean specific humidity (shaded), geopotential height (contours, in 10 m intervals), and wind (arrows), and (d) 850 hPa trends in specific humidity (shaded), geopotential height (contours, in 1 m decade^{-1} intervals, red/blue contours denote positive/negative geopotential height trends), and wind (arrows, statistically significant at 95%). Grid points with a statistical significance exceeding the 95% confidence interval are marked by (a) pink hashes for temperature and blue hashes for MSLP, (b,d) purple hashes for specific humidity. Areas with mean surface pressure below 850 hPa are masked out in (c,d).

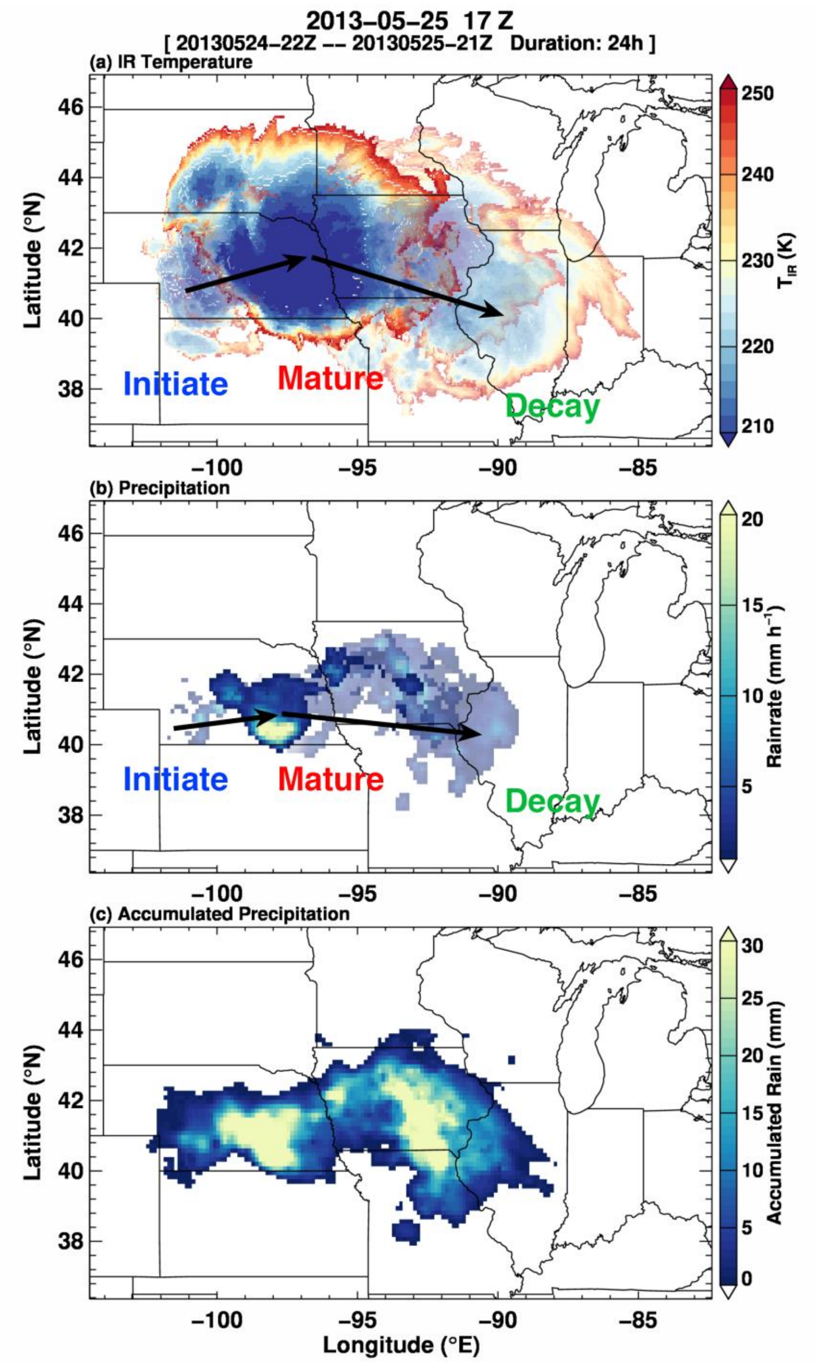

Supplementary Figure 5. Example of a mesoscale convective system tracked by the satellite algorithm. Several snapshots of the mesoscale convective system (MCS) life cycle stages are shown in the (a) satellite infrared (IR) temperature data, (b) NASA North American Land Data Assimilation System (NLDAS) precipitation, and (c) accumulated precipitation over the entire lifetime of the MCS. Arrows in (a,b) show the propagation direction of the MCS.

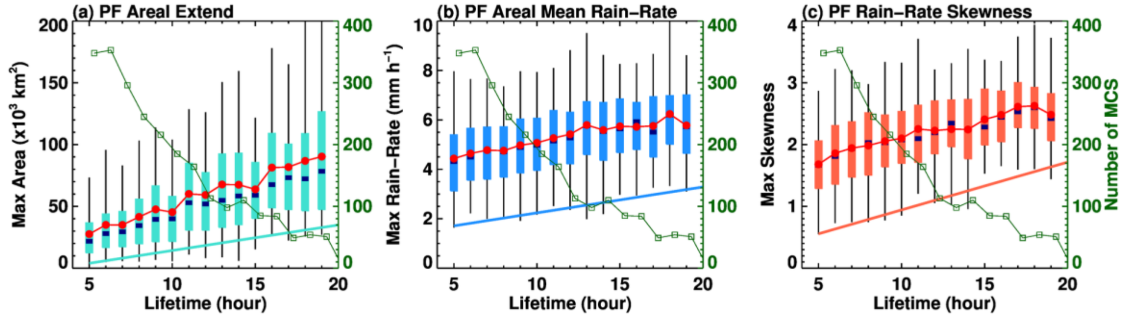

Supplementary Figure 6. Satellite identified mesoscale convective system Precipitation Feature characteristics. Mesoscale convective system (MCS) Precipitation Feature (PF) (a) maximum areal extent, (b) maximum areal mean rain-rate, and (c) maximum rain-rate skewness as a function of MCS lifetime. The MCS database in this case is obtained from satellite infrared (IR) temperature data. Boxes show the area between the 25th and 75th percentiles, whiskers denote the 5th and 95th percentiles, and horizontal bars represent median values. Mean values are shown by the solid red lines with filled circles. The least square fits giving the highest skill score between the PF and satellite MCS database (see text for more details) are shown in the thick solid color lines of each panel. The number of MCSs in each lifetime bin is shown in the green lines with open squares.

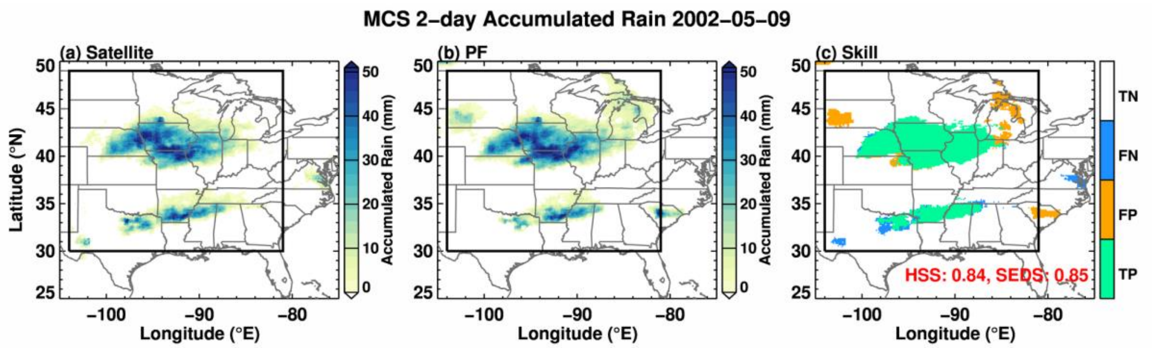

Supplementary Figure 7. Example of 2-day accumulated mesoscale convective systems rainfall identified by the Satellite and Precipitation Feature method. (a) Satellite identified mesoscale convective systems (MCSs), (b) Precipitation Feature (PF) identified MCSs, (c) contingency table, TP: true positive, FP: false positive, FN: false negative: TN: true negative. Pixels with accumulated rainfall > 10 mm are used as a mask to compute skill scores. The Heidke Skill Score (HSS) and Symmetric Extreme Dependency Score (SEDS) are 0.84 and 0.85 for this event. Note that a perfect skill score is 1.

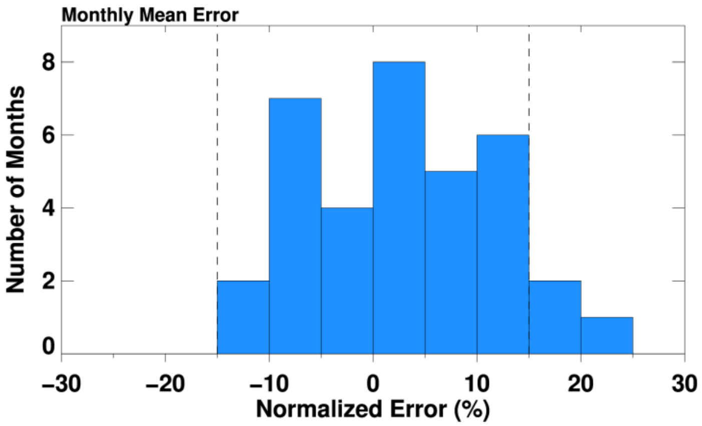

Supplementary Figure 8. Normalized monthly mean precipitation error between the Precipitation Feature identified and reference satellite data identified mesoscale convective systems.

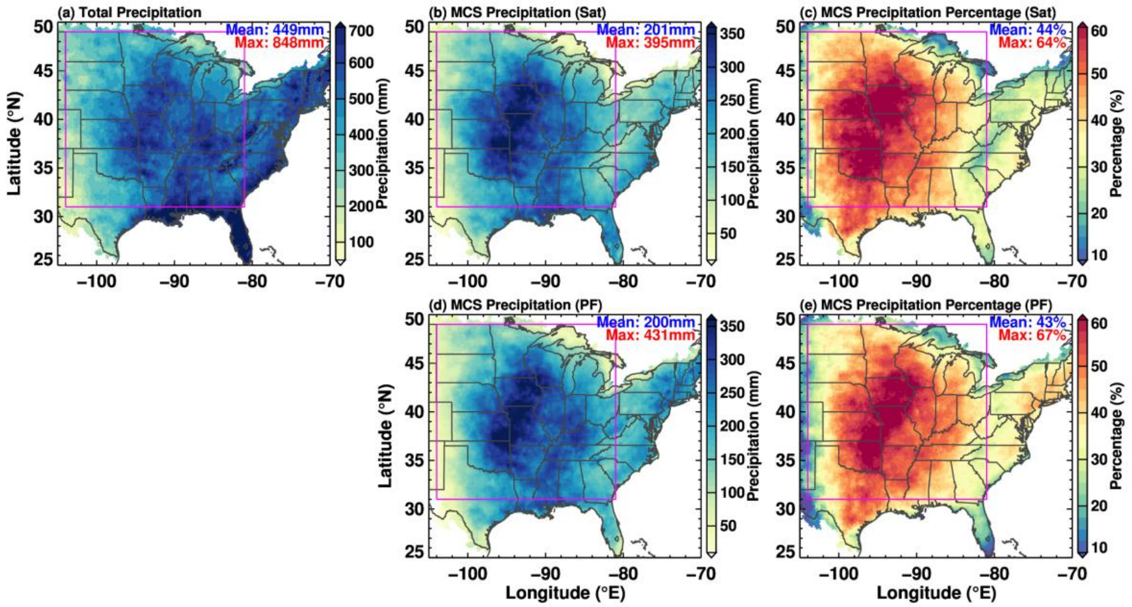

Supplementary Figure 9. 2000-2014 April-August averaged precipitation climatology. (a) Total precipitation, (b) satellite identified mesoscale convective system (MCS) precipitation, (c) percentage of total rainfall from satellite identified MCS, (d) Precipitation Feature (PF) identified MCS, (e) percentage of total rainfall from PF identified MCS. The mean and maximum values in the legends are calculated within the magenta box area.

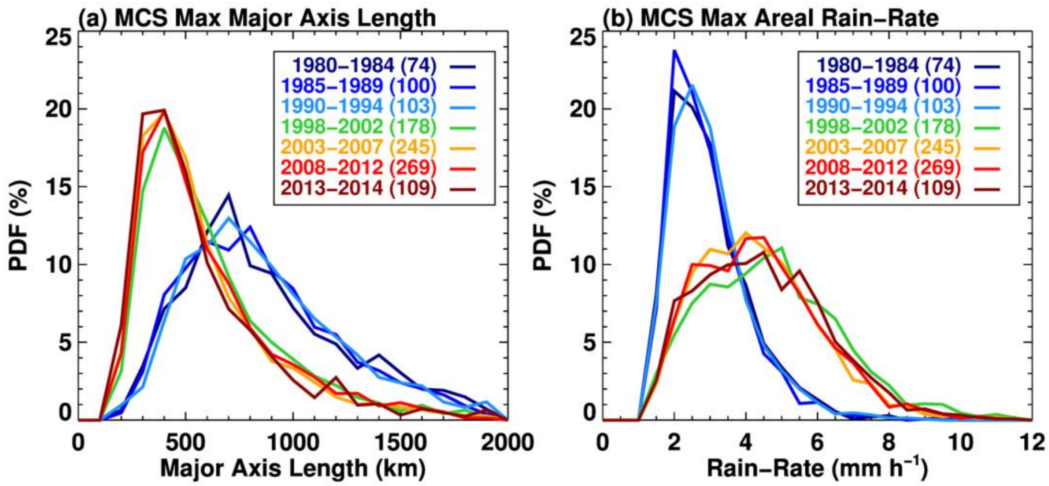

Supplementary Figure 10. Comparison of mesoscale convective system characteristics before and after dataset change around 1996. Probability Distribution Functions (PDFs) of mesoscale convective system (MCS) Precipitation Feature (a) maximum major axis length and (b) maximum areal mean rain-rate using parameters derived from 2000-2014 (Supplementary Figure 5). Each color line is obtained from statistics of 5 warm-seasons with the exception of 2013-2014. The parentheses in the legend show the number of MCSs in each period.

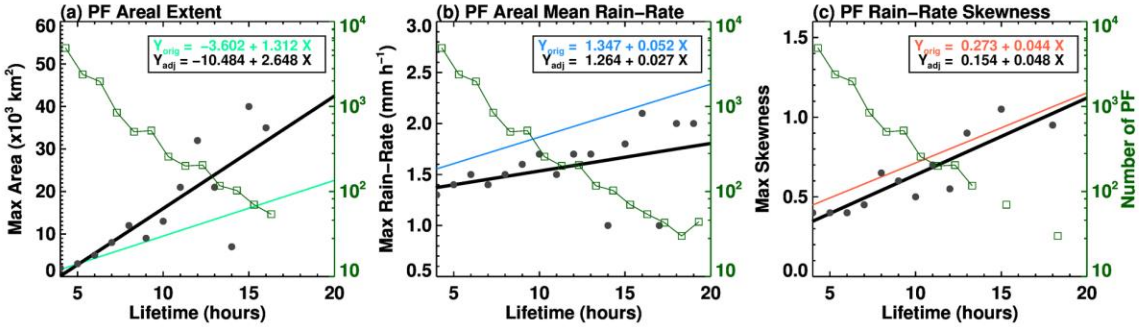

Supplementary Figure 11. Adjusted Precipitation Feature parameters used to identify mesoscale convective systems prior to 1996. Mesoscale convective system (MCS) Precipitation Feature (PF) (a) maximum areal extent, (b) maximum areal mean rain-rate, and (c) maximum rain-rate skewness. The black dots are the 1991-1995 equivalent values for each MCS lifetime derived from PF statistics from 1996-2000. The green lines with open squares are the number of MCSs in each lifetime bin. Lifetime bins with less than 10 PF samples are excluded. The color lines are the same as in Supplementary Figure 5 and the black lines are the new adjusted fit lines.
